# Complete Chloroplast Genome Sequence Of *Prunus Davidiana* (Rosaceae)

**DOI:** 10.1080/23802359.2018.1501325

**Published:** 2018-08-10

**Authors:** Xin Zhang, Jie Yan, Qin Ling, Lu Fan, Manrang Zhang

**Affiliations:** College of Horticulture, Northwest A&F University, Yangling, China

**Keywords:** *Prunus davidiana*, chloroplast genome, Rosaceae, phylogenetic analysis

## Abstract

*Prunus davidiana* is a Chinese wild peach species belonging to the family Rosaceae. In this study, we first assembled the complete chloroplast (cp) genome of *P. davidiana* by Illumina paired-end reads data, then carried out phylogenetic analysis. The *P. davidiana* cp genome was 158,055 bp, containing a large single copy region (LSC) of 86,248 bp and a small single copy region (SSC) of 19,047 bp separated by a pair of inverted repeats (IRs) of 26,380 bp. The cp genome contained 131 genes, including 86 protein-coding genes, 37 transfer RNA (tRNA) genes and 8 ribosomal RNA (rRNA) genes. The result of the phylogenetic analysis showed that *P. davidiana* is closely related to *P. mongolica*.

*Prunus davidiana* is a wild species, belonging to the subfamily Prunoideae of the family Rosaceae. It is native to China and is naturally distributed in most of the provinces of China and exists in various habitats (Leng and Qi 2003). *Prunus davidiana* possesses the advantages of highly drought-resistant and survival rates (Dang et al. 2017), therefore it is very suitable for extensive cultivation and also can reduce the rate of soil erosion in ecological restoration region (Liu et al. 2018). *Prunus davidiana* is related to the cultivated peach, and is a source of abundant resistance to insect pests and diseases (Smykov et al. 1982). Accordingly, this plant has the potential to be used as an advantage traits gene donor source which can be of benefit for future study in peach breeding programs. In this paper, we characterized the complete chloroplast genome sequence of *P. davidiana,* to contribute in further population genetic studies of the genus Prunus of the family Rosaceae.

Fresh leaves of *P. davidiana* were collected from Yulin, Shaanxi, China (37°51'N, 110°23'E). A voucher specimen (AF-06-22) was stored in the Institute of College of Horticulture, Northwest A&F University, Yangling, China. Genomic DNA was isolated with the CTAB method (Doyle and Doyle 1987). The obtained chloroplast DNA was sequenced using Illumina HiSeq 2500 Sequencing system (Illumina, CA, USA) and sequences were used for constructing library. In total, 14,666,376 of 150 bp raw paired-end reads were obtained. The program MITObim v1.8 (Hahn et al. 2013) was employed for the assembly of chloroplast genome, with that of *Prunus persica* (GenBank accession HQ336405) as the reference. Annotation was performed with Dual Organellar GenoMe Annotator (DOGMA) (Wyman et al. 2004), and was manually inspected to predict PCGs, transfer RNA (tRNA) genes, and ribosomal RNA (rRNA) genes. The complete cpDNA sequence of *P. davidiana* has been submitted to GenBank with the accession number MH460864.

The complete chloroplast genome of *P. davidiana* was 158,055 bp in length, consisting of a pair of IR regions (26,380 bp), a small single copy (SSC) region (19,047 bp) and a large single copy (LSC) region (86,248 bp). The chloroplast genome of *P. davidiana* contained 131 genes including 86 protein-coding genes, 37 tRNA genes and 8 rRNA genes. Most of these genes occurred as a single copy, however, eight protein-coding genes (*ndhB, rpl2, rpl23, rps7, rps12, rps19, ycf1, ycf2*), seven tRNA genes (*trnA-UGC, trnI-CAU, trnI-GAU, trnL-CAA, trnN-GUU, trnR-ACG, trnV-GAC*) and four rRNA genes (*4.5S, 5S, 16S, 23S*) in the IR regions are totally duplicated. There were fifteen genes (*atpF, ndhA, ndhB, petB, petD, rpl2, rpl16, rpoC1, rps16, trnA-UGC, trnG-UCC, trnI-GAU, trnK-UUU, trnL-UAA, trnV-UAC*) containing one intron and three genes (*ycf3, clpP, rps12*) containing two introns. The overall GC content of *P. davidiana* chloroplast genome is 36.8% and the corresponding values in LSC, SSC, and IR regions are 34.6%, 30.3%, and 42.6%, respectively. The maximum likelihood (ML) tree showed that *P. davidiana* is closely related to *P. mongolica* (Figure 1).

**Figure 1.**
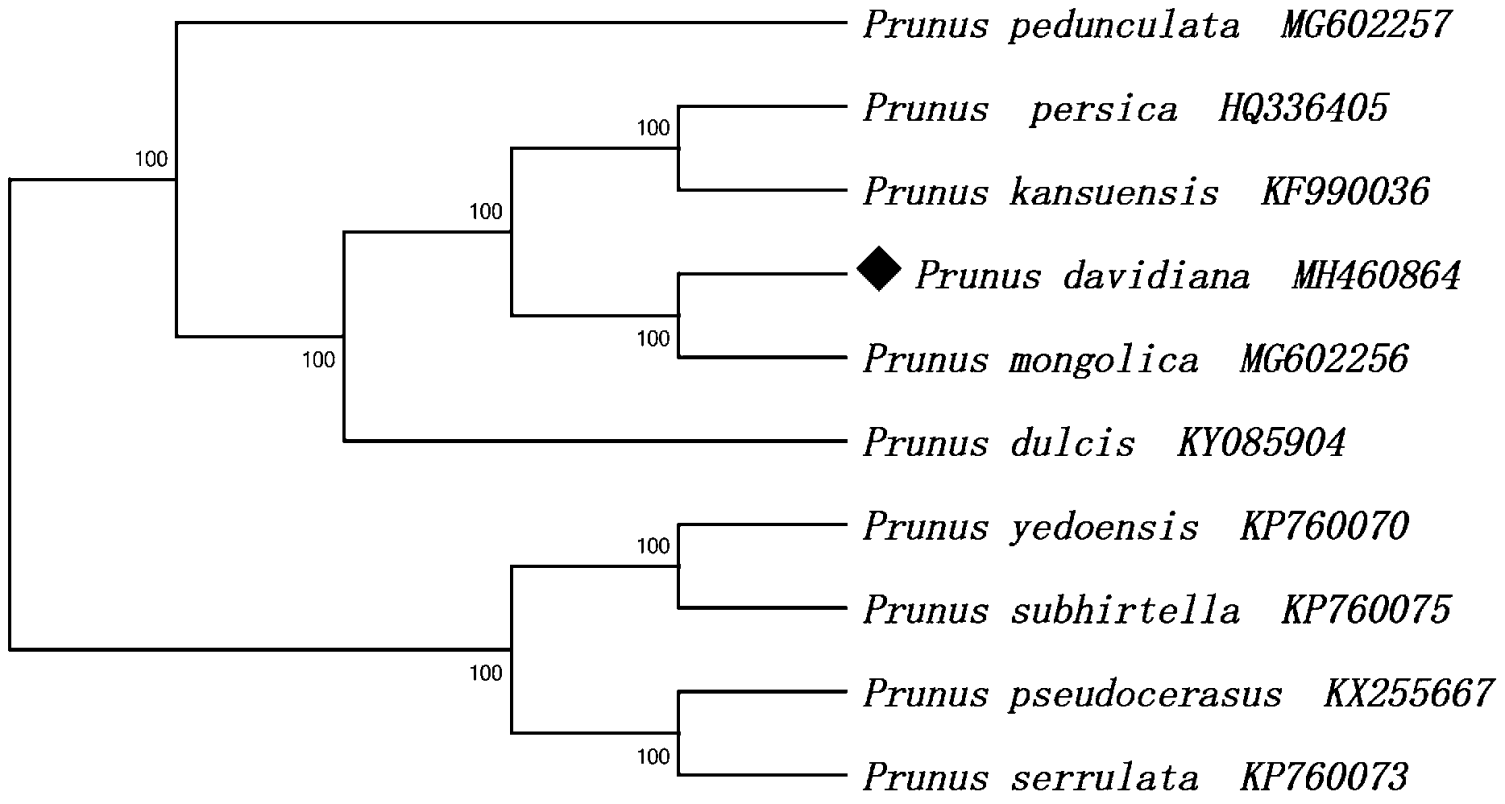
Maximum likelihood phylogenetic tree based on 10 complete chloroplast genome sequences of Rosaceae family. Numbers in the nodes indicate the bootstrap support values from 1000 replicates.
